# A Parallel Evolutionary Computing-Embodied Artificial Neural Network Applied to Non-Intrusive Load Monitoring for Demand-Side Management in a Smart Home: Towards Deep Learning

**DOI:** 10.3390/s20061649

**Published:** 2020-03-16

**Authors:** Yu-Hsiu Lin

**Affiliations:** Department of Electrical Engineering, Ming Chi University of Technology, New Taipei City 24301, Taiwan; yhlin@mail.mcut.edu.tw; Tel.: +886-2-2908-9899 (ext. 4829)

**Keywords:** artificial intelligence, home energy management system, nonintrusive load monitoring, smart home, smart grid

## Abstract

Non-intrusive load monitoring (NILM) is a cost-effective approach that electrical appliances are identified from aggregated whole-field electrical signals, according to their extracted electrical characteristics, with no need to intrusively deploy smart power meters (power plugs) installed for individual monitored electrical appliances in a practical field of interest. This work addresses NILM by a parallel Genetic Algorithm (GA)-embodied Artificial Neural Network (ANN) for Demand-Side Management (DSM) in a smart home. An ANN’s performance in terms of classification accuracy depends on its training algorithm. Additionally, training an ANN/deep NN learning from massive training samples is extremely computationally intensive. Therefore, in this work, a parallel GA has been conducted and used to integrate meta-heuristics (evolutionary computing) with an ANN (neurocomputing) considering its evolution in a parallel execution relating to load disaggregation in a Home Energy Management System (HEMS) deployed in a real residential field. The parallel GA that involves iterations to excessively cost its execution time for evolving an ANN learning model from massive training samples to NILM in the HEMS and works in a divide-and-conquer manner that can exploit massively parallel computing for evolving an ANN and, thus, reduce execution time drastically. This work confirms the feasibility and effectiveness of the parallel GA-embodied ANN applied to NILM in the HEMS for DSM.

## 1. Introduction

Electricity is one of the most popular forms of energy used in today’s technically-driven modern society. The reformation of a traditional power grid upgraded as a smart grid has been developed in recent years. In a smart grid, electrical energy demands by consumers are continuously increasing. To meet continuously increasing electrical energy demands, power utilities come up with Demand Response (DR) programs, which is referred to as Demand-Side Management (DSM). In DSM, traditionally, Energy Management Systems (EMSs) that respond to DR programs to optimize consumers’ uses of electrical energy so that continuously increasing electrical energy demands by consumers can be met monitor electrical appliances with an intrusive deployment of smart power meters (smart plugs) in practical fields of interest. However, constructing such an EMS in a practical field of interest for DSM would result in a high investment, which comprises annual maintenance costs, to consumers. In contrast to intrusive load monitoring in EMSs for DSM, technically, Non-Intrusive Load Monitoring (NILM) being a part of an EMS in a practical field of interest for DSM is a process of decomposing mains’ electricity measurements into each of their constituent individuals with distinguishable electrical characteristics from electrical appliances, where aggregated electric energy consumption is measured by only one single minimal set of plug-panel current and voltage sensors in a practical field of interest and is decomposed for its breakdown from appliance-level electric energy consumption. NILM, which is a cost-effective alternative to intrusive load monitoring in EMSs, was first investigated by George W. Hart et al. [1] at the Massachusetts Institute of Technology with funding from the Electric Power Research Institute in the early 1980s. NILM has been conducted for DSM in a smart grid [2,3]. With the use of NILM for DSM in a smart grid, consumers have the opportunity to improve their awareness of what and when their electrical appliances should operate in response to DR programs [4], where they keep track of appliance-level electric energy consumption with no intrusive deployment of smart power meters (smart plugs) for individual electrical appliances. With DSM in a smart grid, power utilities plan to supply electricity in an optimal way [5].

NILM conducted in EMSs for DSM in a smart grid can be built upon signal processing [6], data clustering and pattern recognition (a.k.a. machine learning) [2,3,7,8,9,10,11,12,13,14,15,16], and/or Artificial Intelligence (AI) (a.k.a. computational intelligence/deep learning) [17,18,19,20,21]. NILM can be viewed as a load classification task, and it can be accomplished by Artificial Neural Networks (ANNs). For example, in References [20,21], feed-forward ANNs trained through the conventional gradient-based optimization methods were conducted and used as load classifiers of NILM to non-intrusively identify electrical appliances. ANNs are one of the most promising biologically inspired AI paradigms ever invented. To an ANN used as a load classifier of NILM, its performance in terms of classification accuracy depends on its training algorithm. For instance, a Genetic Algorithm (GA), which is a global search optimization algorithm starting with an initial population of candidate solutions, instead of the widely-used Gradient Descent (GD) algorithm, a conventional gradient-based optimization algorithm starting with an initial single guess and taking derivatives for its steps that are proportional to the negative of the gradient of the cost function addressed, can be used to meta-heuristically train/evolve the network (the network trained by the conventional GD algorithm is easily fooled by local minima). Nevertheless, meta-heuristically training/evolving an ANN/deep NN learning from massive training samples is extremely computationally intensive since GAs are extremely computationally intensive routines because they search through a large set of candidate solutions involving expensive function evaluations to find the global (quasi-)optimal solution, whereby the sequential optimization process could consume many hours or days. Parallel computing is able to improve the computational efficiency of GAs by exploiting the concurrency of computations involved in the algorithm. Therefore, the aim of this work is to integrate meta-heuristics (evolutionary computing) with an ANN (neurocomputing) considering its evolution in a parallel fashion for load disaggregation in a home EMS (HEMS). A parallel GA-embodied ANN for NILM in an HEMS is developed in this work. To parallelize and, thus, accelerate a GA incorporated with an ANN and used to evolve/train the connectionism in parallel for NILM is done in this work. The GA-embodied ANN in this work is processed and accelerated in consideration of parallel computing. The use of the highly-parallelized GA run on multiple processors is mandatory to achieve high-performance computing for ANN evolved/meta-heuristically trained for NILM. NILM can be applied in prognostics and health management where untypical patterns of electric energy consumption may be caused by the malfunction of an electrical appliance(s) [22].The major contributions of this work are outlined as follows.

- In contrast to the work that was finished in References [12,20,21], this work develops and applies a parallel GA to meta-heuristically evolve a feed-forward ANN, in order to achieve its optimal adjustable parameters, optimal trainable weight coefficients including biases, and speed up the evolutionary process for meta-heuristically training the connectionism for load classification in NILM conducted as a part of a Home Energy Management System (HEMS) for Demand-Side Management (DSM). The developed parallel GA-embodied ANN is a revision of the methodology that was done in Reference [12]. In addition, it is a preliminary design of NILM toward deep learning.
- NILM addressed in this work is a fully-nonintrusive alternative that, during the preliminary stage of NILM involving the training process of load classification, no instrumentation for (rated) power consumption on each electrical appliance is required. It is suggested that, in the preliminary stage of NILM, collected data that need to be learned for load classification can be labeled in a sensor fusion fashion. Through the NILM with respect to aggregated electric energy consumption, electrical appliances for their electric power consumption can be deduced/statistically estimated.

The remainder of this work is organized as follows. The methodology of the parallel GA-embodied ANN applied to NILM in an HEMS is presented in Section 2. Section 3 demonstrates the experiment showing the feasibility of the parallel GA-embodied ANN for NILM in this work. The experiment is conducted in a real residential environment. Section 4 makes some discussion on the experiment demonstrated in Section 3, which explores the significance of the experimental results of this work. Lastly, this work is concluded with its future work in Section 5.

## 2. Methodology

The developed parallel GA-embodied ANN applied to NILM in an HEMS is presented in this section. Section 2.1 presents the HEMS deployed in a real residential field for the demonstration of the developed parallel GA-embodied ANN for NILM. Section 2.2 presents the used ANN and Genetic Algorithm (GA) hybridized to evolve the connectionism in parallel.

### 2.1. HEMS

**Figure 1:**
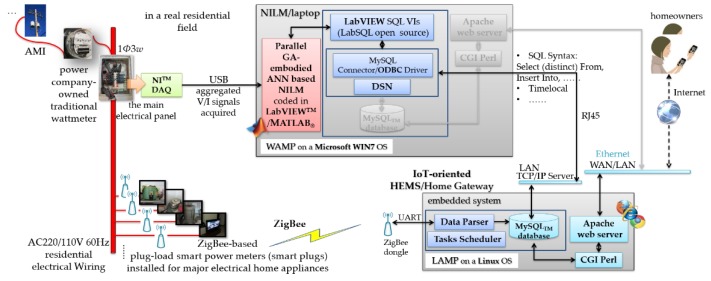
Block diagram of the home energy management system (HEMS) [24] having the developed parallel GA-embodied artificial neural network (ANN) for non-intrusive load monitoring (NILM).

An HEMS conducted in a smart home in a smart grid works as a bi-directional communication interface between a residential field, which is aware of DR programs, and a power utility, which analyzes electric power consumption data from grid-tied smart homes, comes up with DR programs, and optimizes consumers’ uses of electrical energy [23]. A typical HEMS developed and deployed in a smart home for DSM in a smart grid can be mainly made of (i) a power company-owned smart meter installed instead of a power company-owned traditional wattmeter, communicated via advanced metering infrastructure with a power utility’s cloud server, and used to continually gather fine-grained electric power consumption data from electrical appliances, (ii) a central Home Gateway (HG) communicated via the Internet with a third-party cloud server for user-centric IoT service-oriented applications and with the power utility’s cloud server for IP-based DR programs received to homeowners for their load management, and (iii) electrical appliances monitored. Figure 1 shows the block diagram of the HEMS [24] with the parallel GA-embodied ANN developed in this work for NILM. In Figure 1, the HEMS with the developed parallel GA-embodied ANN for NILM was deployed in a residential field, which is connected with a smart grid for DSM. Since the goal of DSM in a smart grid is to optimize consumers’ uses of electric power consumption by electrical appliances in fields of interest, the HEMS with the developed parallel GA-embodied ANN for NILM identifies electrical appliances.

As shown in Figure 1, the HG, the core of the HEMS, is based on an Advanced RISC Machine (ARM^®^) Cortex^TM^-A9 embedded system. Moreover, a LAMP (a Linux OS + an Apache^®^ HTTP server + a MySQL_TM_ relational database + PHP scripting language) stack is configured on it. In the residential field, the HEMS with the developed parallel GA-embodied ANN for NILM is demonstrated. Wherein, the HEMS continually acquires power consumption on each of its monitored electrical appliances in an intrusive manner, whereas the developed parallel GA-embodied ANN identifies it in a non-intrusive fashion. Data from either the HEMS or the developed parallel GA-embodied ANN for NILM are stored in the MySQL_TM_ relational database configured on the HG. The developed parallel GA-embodied ANN for NILM is implemented in MATLAB^®^ and run on a computer. In the NILM communicated with the HEMS, a standardized *MySQL Connector/Open Database Connectivity (ODBC)* driver and *LabSQL* Virtual Instruments using the ADO (ActiveX Data Objects) object collection and SQL (Structured Query Language) in LabVIEW^TM^ are included. ZigBee (the IEEE 802.15.4 standard) [25] is arguably the most popular technology for creating wireless sensor networks, and it has been included in some of the latest commercial and academic home automation cases [26,27,28,29].

By means of a ZigBee wireless communication network built in the HEMS in Figure 1, the HG is capable of communicating with its monitored electrical appliances for intrusive load monitoring and remote load control. ZigBee communication-based plug-load smart power meters (smart plugs) are installed for DSM. Universal Asynchronous Receiver/Transmitter (UART), which is a RS232 RTU-format protocol, is used in the HEMS. Lastly, as shown in Figure 1, in the HEMS, Common Gateway Interface (CGI) is programmed in Perl programming language and configured on the Home Gateway (HG). Additionally, an HTTP web server is established, where homeowners can interact dynamically with the HEMS, via the Internet, using a user interface showing detailed electric power consumption data. Compared to the HEMS [24] as a benchmark, the NILM identifies electrical appliances with no intrusive deployment of ZigBee communication-based plug-load smart power meters (smart plugs) in the residential field. The assumption made in Reference [24] is that each electrical appliance enrolled for DR programs and monitored in the residential field is equipped with a wireless remote controller such as an infrared (IR) controller [12]. Each electrical appliance is remotely controllable (its electric power consumption is deduced through NILM). In this sense, in Figure 1, aggregated electric energy consumption measured by the one single minimal set of plug-panel current and voltage sensors (installed at the main electrical panel of the residential field) is labeled through sensor fusion and identified by the developed parallel GA-embodied ANN for NILM in this work. Wi-Fi flows interpreting occupants’ energy-use patterns [30] in a practical field of interest could also be utilized to label aggregated electric energy consumption through sensor fusion for NILM. The developed parallel GA-embodied ANN for NILM is presented in Section 2.2.

### 2.2. NILM Based on a Parallel GA-Embodied ANN to Fully-Nonintrusively Identify Electrical Appliances Consuming Electric Power

NILM is a load disaggregation technique that aims to identify electrical appliances as well as estimate their power consumption from aggregated electric energy consumption by multiple electrical appliances [18], which can be defined by Equation (1).

(1) $$y_{t}=\sum_{i=1}^{N}x_{t}^{i}\cdot P_{i}+\varepsilon_{t}$$

where *y_t_* represents the aggregated observable power consumption by all electrical appliances at time slice *t* in a practical field of interest, *N* is the total number of major electrical appliances, *x^i^_t_* denotes the operational state, {0, 1} in one mode, of electrical appliance *i* at time slice *t* with its rated power consumption *P_i_*, and *ε_t_* accounts for total power consumption by small loads at time slice *t*, which is treated as background noise.

The goal of NILM/load disaggregation in Equation (1) is to build an Artificial Intelligence (AI) model that can find the correct *x^i^_t_* from *y_t_*. In this work, the AI model is constructed with a feed-forward ANN meta-heuristically trained by a parallel Genetic Algorithm (GA).

**Figure 2:**
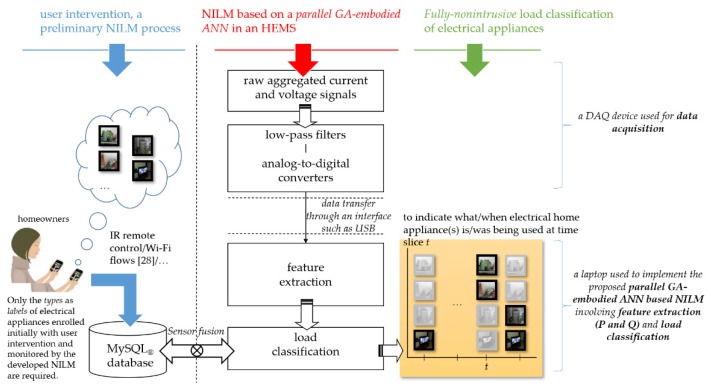
Workflow of the non-intrusive load monitoring (NILM) using a parallel genetic algorithm (GA)-embodied Artificial Neural Networks (ANN) for NILM in this work. The NILM is fully-nonintrusive that no instrumentation for (rated) power consumption on electrical appliances is required during the preliminary stage of the NILM involving the training process of the ANN for load classification.

The workflow of the NILM utilizing the developed parallel GA-embodied ANN in this work is depicted in Figure 2. The NILM consists of data acquisition that acquires electrical signals from one single minimal set of plug-panel current and voltage sensors, feature extraction that extracts electrical features for load classification (in feature extraction, more technically, feature data can be transformed from a data space to a feature space for dimensionality reduction), and load classification that conducts AI to identify electrical appliances from feature data (electric power consumption on electrical appliances and electrical energy demands by homeowners can be deduced). Both aggregated current and voltage signals are simultaneously acquired via data acquisition where signals sensed by one single minimal set of plug-panel current and voltage sensors are conditioned and digitized by a data acquisition device. After aggregated current and voltage signals are sampled, sampled data are analyzed through feature extraction and load classification. A common NILM framework, known as event-based NILM, involves a process of event detection that detects appliance events by abrupt power changes for feature extraction and load classification. In the NILM in Figure 2 in this work, the process of feature extraction and load classification is performed periodically. Electrical appliances are identified every time slice *t*. The NILM in this work is an eventless NILM. In practice, an AI-based paradigm in NILM is developed where (i) its training stage is intrusive as fields of interest are instrumented for (rated) power consumption on each of electrical appliances and (ii) its test/validation stage is not intrusive as no instrumentation for (rated) power consumption on each electrical appliance is required (plug-load smart power meters (smart plugs) used in the training stage of NILM are removed). The NILM in Figure 2 is a fully-nonintrusive alternative that, during the preliminary stage of the NILM involving the training process of the ANN for load classification, no instrumentation for (rated) power consumption on each electrical appliance is required (only the types as labels of electrical appliances enrolled initially with user intervention and monitored by the NILM are required). Electrical appliances are labeled through sensor fusion [30] (electrical appliances can be deduced/statistically estimated, for their electric power consumption, through the NILM with respect to aggregated electric energy consumption).

**Figure 3:**
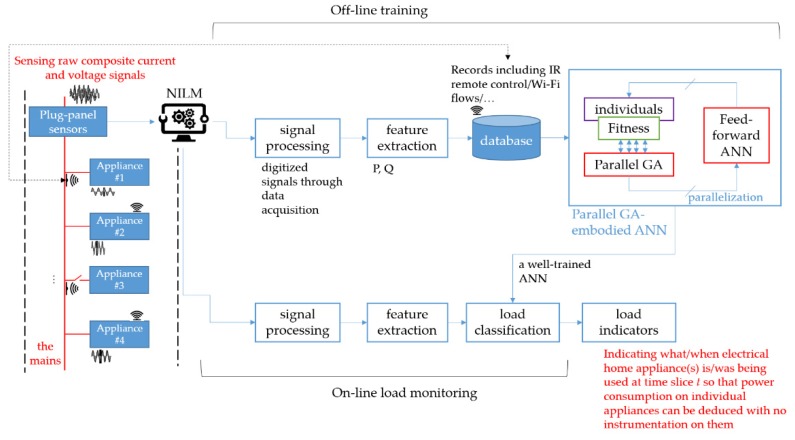
A detailed NILM pipeline in this work.

Figure 3 also depicts the detailed NILM pipeline proposed and referenced during the experiment in this work. In the NILM in Figure 2 and Figure 3, electrical features extracted from acquired and aggregated current and voltage signals in feature extraction and used as the input variables of the feed-forward ANN in load classification are steady-state electric power quantities P (real power) and Q (reactive power) [31]. This is contrary to steady-state electrical features. Transient electrical features are electrical features by an event-based NILM with event detection and feature extraction [31,32]. The feed-forward ANN identifies electrical appliances based on their steady-state behavior. In this work, P and Q are considered, as an NILM considering only P cannot distinguish electrical appliances with similar P draws (additionally, transient electrical features extracted can be used as additional/supplementary electrical features [31,32]). In the NILM in Figure 2 and Figure 3, load classification is implemented by a feed-forward ANN, which is a multi-layer perceptron. The feed-forward ANN conducted in the NILM learns from training samples collected on-site during the training stage of the connectionism. The training stage of the connectionism is an off-line training process, where (1) collected data are labeled through sensor fusion and (2) the connectionism is evolved by a parallel GA. On-line load classification is performed on-site (every time slice *t*) once the connectionism has been well trained. The preliminaries regarding ANNs and GAs are outlined in Section 2.2.1 and Section 2.2.2. The developed parallel GA-embodied ANN in Figure 3 is presented in Section 2.2.3 for NILM in this work.

#### 2.2.1. ANN

**Figure 4:**
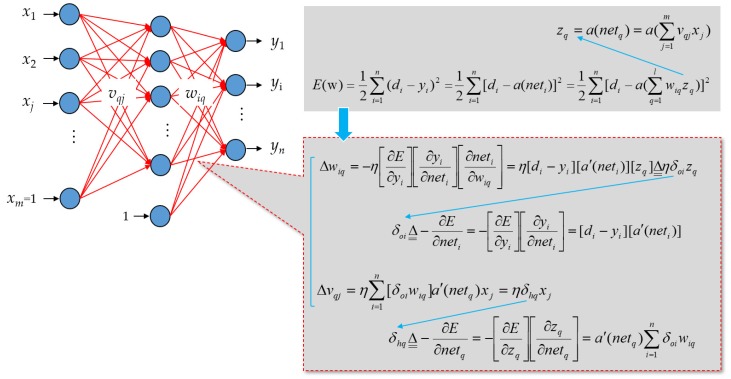
A standard feed-forward and multi-layer ANN as a connectionism mimicking the biologic neural network and consisting of an input layer including at least one hidden layer and an output layer. Usually, the more hidden artificial neurons the neural network has, the more accurate the neural network is.

In general, solutions of many engineering problems are similar to a black box, which is an opaque system used to empirically establish the relationship between input and output variables without the knowledge of its internal working model. ANNs date back to 1943. A mathematical model of artificial neurons was proposed in Reference [33]. ANNs, which are connectionist systems, are one of the approaches that can empirically establish the relationship between input and output variables without the knowledge of its internal working model. ANNs are inspired by biological neural networks that constitute animal brains. They are one of the most promising biologically inspired AI paradigms ever invented. In this work, a standard feed-forward ANN is employed. Figure 4 depicts the employed standard feed-forward three-layered ANN. For the topological design of the network, its architecture by the hyperparameters, which are the number of hidden layers and the number of artificial neurons conducted in each hidden layer, can be determined based on trial and error, which can also be determined through some heuristics [12]. Usually, it is true that the more hidden artificial neurons the feed-forward multi-layer ANN has, the more accurate the neural network is [12].

Deep Neural Networks (NNs) having multiple hidden layers [34,35,36] can be employed, over fog-cloud analytics [2,37,38], for NILM in this work. The employed feed-forward ANN, which is structured by three different types of layers including input, hidden, and output layers consists of an interconnected group of artificial neurons with trainable weight coefficients, *v_qj_* and *w_iq_*, in Figure 4. In a feed-forward and multi-layer ANN in Figure 4, the number of artificial neurons in a hidden layer(s) can be determined by the approaches proposed in References [39,40]. A supervised and iterative learning process such as the widely-used GD algorithm [41] that was originally proposed in Reference [42] can be used to train the ANN learning model from collected training samples of input-output data pairs. Once the ANN is well-trained, its weight coefficients are ultimately adjusted such that its outputs against their expected values are satisfied with a satisfactory level of accuracy. The widely-used GD algorithm can be conducted for use of training an ANN with back-propagation. Nevertheless, its performance in terms of classification accuracy depends on the training algorithm adopted. Neuro-evolution is a research field of AI. It conducts evolutionary computing to meta-heuristically render a certain aspect of the design of ANNs designed and used for practical applications. A GA [43,44] briefly introduced in Section 2.2.2 can be used to train an ANN [45]. However, training an ANN/deep NN learning from massive training samples is extremely computationally intensive. Hence, a parallel GA described in Section 2.2.3 is conducted and used to integrate meta-heuristics (evolutionary computing) with an ANN (neurocomputing), the connectionism in Figure 4, considering its evolution in a parallel execution and relating to load classification for NILM in this work. Hybridizing a GA with an ANN is to develop a problem solver for NILM by looking at and mimicking the human brain (neurocomputing) and the evolution mechanism (evolutionary computing), as the human brain, which is the product of millions of years of evolution, is the most useful and powerful problem solver in the universe. A parallel GA instead of a standard GA can exploit massively parallel computing for training an ANN and, thus, reduce computation time drastically.

#### 2.2.2. GA

GAs are based on the principle of biological evolution. They are a stochastic, population-based meta-heuristic optimization algorithm that searches for the global (quasi-)optimal solution randomly, through genetic operations, involving crossovers and mutations that provide genetic diversity, among population members, based on (natural) selection.

**Figure 5:**
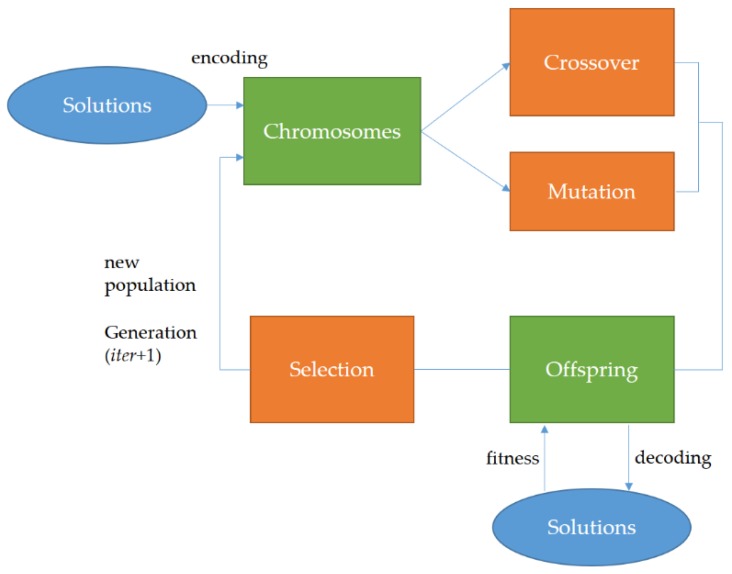
A typical evolutionary cycle of a genetic algorithm (GA).

GAs that mimic the process of biological evolution are proven to be effective in many optimization problems addressed. In GAs, two different types of operations are used: genetic operations and evolution operation. Where genetic operations involve crossover and mutation operations and evolution operation involves selection operations mimicking natural selection, the Darwinism--Darwin’s theory of biological evolution, and being the driving force of evolution. Different selection strategies and genetic operators can be designed and implemented for GAs. Refer to Reference [43] for more details about a standard GA. In a GA used to address an NP-hard/NP-complete discrete or continuous optimization problem, a population of chromosomes (called individuals as candidate solutions in a search space) is evolved through selection, crossover, and mutation operations toward the global (quasi-)optimal solution. Figure 5 shows the typical evolutionary cycle of a GA.

In a GA in Figure 5, a proportion of the existing population in the current generation (generation *iter*) is reproduced and used to breed a new population for the next generation. Where,

- Selection selects chromosomes based on a fitness-based selection/proportional selection procedure and fitter solutions (with relatively high fitness) are typically more likely to be selected (candidate solutions are evaluated through a fitness function),
- crossover exchanges subparts of two chromosomes, roughly mimicking biological recombination between two haploid organisms, and
- mutation randomly alters values of genes in some chromosomes. An arbitrary bit of a chromosome is changed from its original state. In a GA, the population size depends on the nature of the problem addressed, but, typically, the population contains several hundreds or thousands of chromosomes/candidate solutions. Chromosomes are generated at random in the initial population. The generational process is repeated until a termination condition has been met. For example, the generational process will terminate when the pre-specified maximum number of generations is reached.

#### 2.2.3. Parallel Evolutionary Computing-Parallel GA

A GA can be used to meta-heuristically train an ANN, the connectionism in Figure 4, without the use of any gradient information (for the connectionism with continuous activation functions, it is an appropriate compromise to hybridize the meta-heuristics with a gradient method such as the widely-used GD algorithm. An initial genetic search is followed by a gradient method). The basic concept behind this technique is as follows.

**Figure 6:**
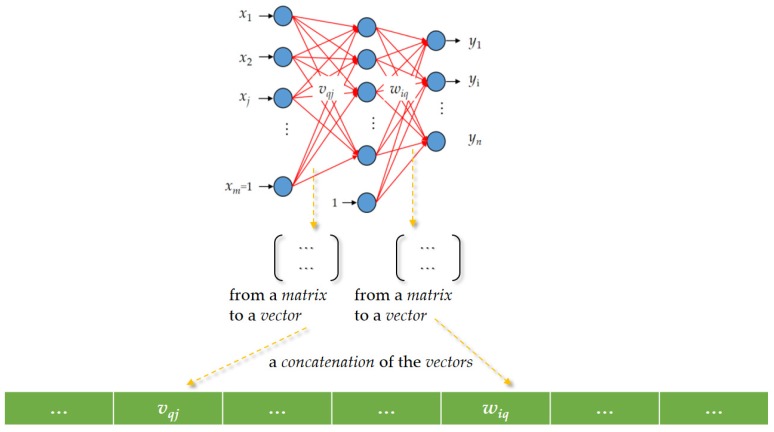
A chromosomal design encoding a feed-forward artificial neural network (ANN) as a chromosome. A chromosome (genotype) completely describes an ANN (phenotype).

A complete set of trainable weight coefficients, *v_qj_* and *w_iq_*, in the connectionism in Figure 4, is encoded in a real-number string, which has an associated fitness indicating its effectiveness. An illustration showing that trainable weight coefficients of an ANN are encoded in order as a chromosome for a genetic search is given in Figure 6. Layered trainable weight coefficients are arranged from a matrix to a vector, and a concatenation of the vectors is built. For a genetic search, fitness can be simply given by –*E*, where *E* is the function value of a cost function for an ANN decoded and evaluated for its trainable weight coefficients. To evaluate a chromosome, the GA decodes it as an ANN (its genes, trainable weight coefficients, are assigned in a network of a given architecture). Then, the decoded ANN is run over collected training samples. Lastly, computed *E* is returned (the decoded ANN plays the role of an evaluation function in the GA). With an initial population of randomly-generated chromosomes started, successive generations are constructed through evolution. Fitter chromosomes are more likely to survive and to participate in genetic operations based on selection. Unlike the widely-used Gradient Descent (GD) algorithm, the GA performs a global search. It is not easily fooled by local minima.

**Figure 7:**
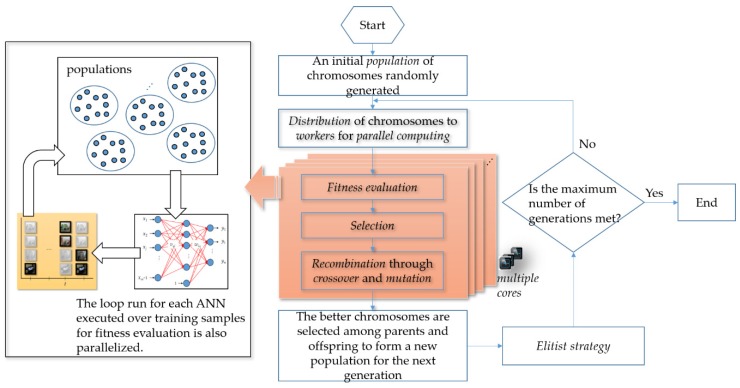
Flowchart of a parallel GA used to evolve an ANN for NILM in this work. In the GA, function evaluations/routines are farmed out to different processors. They are done simultaneously.

Evolving an ANN run over massive training samples for fitness evaluation during evolution is computationally intensive. One of the ways to speed up a GA as a parallel GA is a parallelism to execute evolutionary computing in a parallel execution. A parallel GA is an algorithm that works in a divide-and-conquer manner. Evolving an ANN run over massive training samples for fitness evaluation during evolution can be accelerated by a parallel GA. GAs are innately a parallel algorithm, which can be run on a multi-core processor. For a parallel GA used to evolve an ANN for NILM in this work, the process of fitness evaluation during evolution is distributed among multiple processors as workers. The evaluations of fitness to an architecture-given ANN considering potential weight coefficients are executed, in parallel, across multiple Central Processing Units (CPUs) on a single computer. The flowchart illustrating the computational routines involved in the parallel GA for a multi-core processor/computer is depicted in Figure 7.

In a GA, selection chooses individuals, as parents, to be reproduced for the next population in the next generation. In the parallel GA in Figure 7, a stochastic uniform selection lays out a line in which each parent corresponds to a section of the line of length that is proportional to its scaled fitness value, where a ranking method that scales raw fitness values based on the rank of each individual rather than its raw fitness value is considered for fitness scaling.

In a GA, fitness scaling that converts raw fitness values into scaled ones falling in a range that is suitable for selection is needed because, in a proportional selection procedure, there would be a tendency for a few super chromosomes that dominate the selection procedure to make the algorithm converge prematurely. A practical variant of the reproduction process of constructing a new population is to allow the best haploid organism(s) from the existing population/current generation to carry over to the next population/generation, unaltered. It is known as an elitist strategy, which can be used to guarantee that the solution quality will not decrease during evolution. In the parallel GA in Figure 7, an elitist strategy is considered. In this work, the fitness function is clarified as –*E*, where *E* is the averaged Sum of Squared Errors (SSE) of an architecture-given ANN for NILM. In the algorithm, an ANN with its weight coefficients is drawn from a chromosome, a concatenation of real-valued numbers representing the trainable weight coefficients, for fitness evaluation. Additionally, function evaluations/routines are farmed out to and executed in parallel across multiple CPUs on a single computer. The loop run for each ANN executed over training samples for its averaged SSE/fitness evaluation is also parallelized.

In a GA, crossover is used to combine individuals, who are parents chosen through selection, to form children for the next population in the next generation. In the parallel GA in Figure 7, a scattered crossover operator applied on two parents as two different ANNs for load classification in NILM

**Figure 8:**
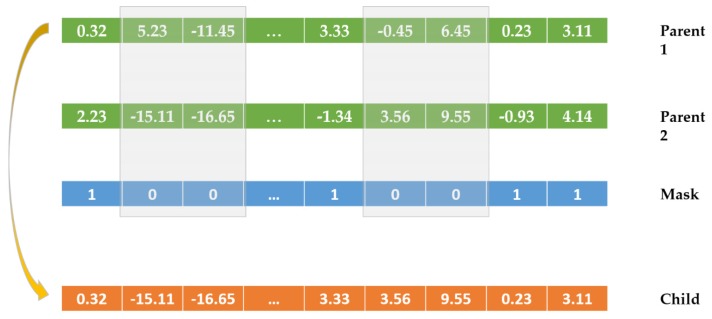
A scattered crossover operator applied on two individuals.

- creates a random binary vector as a mask,
- picks genes from the first parent, where indexing of the created vector is 1s,
- picks genes from the second parent, where indexing of the created vector is 0s, and
- combines the genes to form a new individual as a new/potential ANN for load classification in NILM. The scattered crossover is illustrated in Figure 8.

In a GA, a mutation that creates mutated individuals is used to make small random changes in individuals. In the parallel GA in Figure 7, a gaussian mutation operator applied on an individual replaces a gene value of an individual with a random number taken from a Gaussian distribution where its mean is 0 and its standard deviation is determined by a scale parameter specifying the initial standard deviation and a shrink parameter controlling how the standard deviation shrinks as generations go by.

In Figure 7, the iterative evolutionary cycle of the parallel GA applying the genetic operators after the selection procedure is completed will continue until the maximum number of generations is met. The genetic operators facilitate and guide an efficient GA search where the crossover operator facilitates exploration of the GA search, while the mutation operator facilitates exploitation of the GA search. Additionally, they achieve the twin goal of maintaining population diversity and sustaining the convergence capacity of the algorithm.

## 3. Experiment

**Figure 9:**
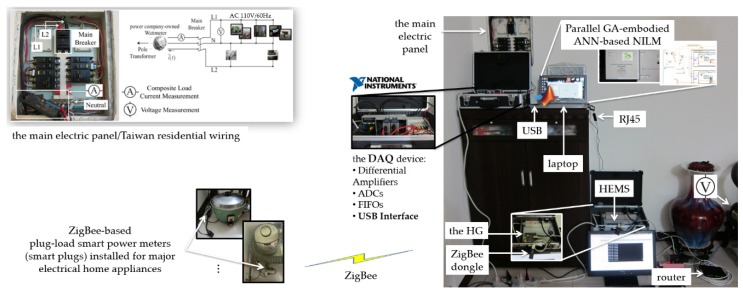
Experimental set-up of the home energy management systems (HEMS) [24] with the NILM in this work.

The experiment conducted to validate the feasibility of the developed parallel GA-embodied ANN for NILM in this work is demonstrated in this section. The HEMS employing the parallel GA-embodied ANN proposed for NILM and presented in Section 2 was deployed in a real residential field. As referenced in Figure 3, Figure 9 shows the experimental setup of the HEMS, as a benchmark/the ground truth, with the NILM. In this experiment, electrical appliances identified by the developed parallel GA-embodied ANN for NILM in Line Branch 1 (L1) of the electrical wiring in the residential field include an electric rice cooker (~1.10 kW), an electric water boiler (~0.90 kW), a steamer (~0.80 kW), and a TV (~0.22 kW). For the HEMS [24] that intrusively identified the electrical appliances in the residential field, ZigBee communication-based plug-load smart power meters (smart plugs) were installed. For the NILM, in this work, that non-intrusively identifies the electrical appliances in the residential field, the aggregated current and voltage signals were acquired and collected from the main electric panel of the residential field by a National Instruments (NI)^TM^ 9225 data acquisition device. An active current transformer that generates output voltage that is proportional to its input current was connected to L1. Its output voltage was wired to the data acquisition device. The electricity was also directly wired to the data acquisition device. The computer used to run the NILM developed in this work, presented in Section 2.2, and implemented in MATLAB^®^ is an Acer Predator G3-710 Intel^®^ Core^TM^ i7-6700 CPU (@ 3.40GHz) quad-core desktop computer where the memory size, RAM, is 16GB. At each time slice, such as *t* = 1 min., taking an electrical feature reading, the NILM identifies the electrical appliances, where a feed-forward ANN hybridized with a parallel GA is used. The feed-forward ANN hybridized with a parallel GA is configured as follows.

- Input layer: the total number of artificial neurons in the input layer of the connectionism is 2 due to the two extracted electrical features, P and Q. Each artificial neuron in this layer comes with an identity-style activation function. Training samples conveyed to the connectionism are normalized within the interval [−1, 1].
- Hidden layer: only one single hidden layer is considered. It consists of 25 artificial neurons. Each artificial neuron in this layer is aroused with a sigmoid-style activation function. That is, artificial neurons used in this layer are the most basic computational sigmoid neurons [46,47] (there are many other nonlinear formulas, nonlinearities, that can be implemented as nonlinear activation functions for feed-forward multi-layer ANNs). In deep NNs conducting a many-layered structure of hidden layers, one shortcoming of the sigmoid neurons is the gradient vanishing problem [46,47,48].
- Output layer: the total number of artificial neurons in the output layer of the connectionism is 4 due to the four electrical appliances monitored. Each artificial neuron in this layer serves as an appliance indicator that the operational ON/OFF status of the electrical appliances, at time slice *t*, is indicated. Its output is based on the round() function rounding its input argument to the nearest integer 0 (indicating status *OFF*) or 1 (indicating status *ON*). The style of the activation functions used by the artificial neurons in this layer is the same as that of the activation functions used by the artificial neurons in the hidden layer.
- Cost function: averaged Sum of Squared Errors (SSE) is adopted. In this experiment, a parallel GA instead of the widely-used GD algorithm is conducted and used to train the configured feed-forward ANN/connectionism. The configured feed-forward ANN is evolved, which is parallelized.

**Figure 10:**
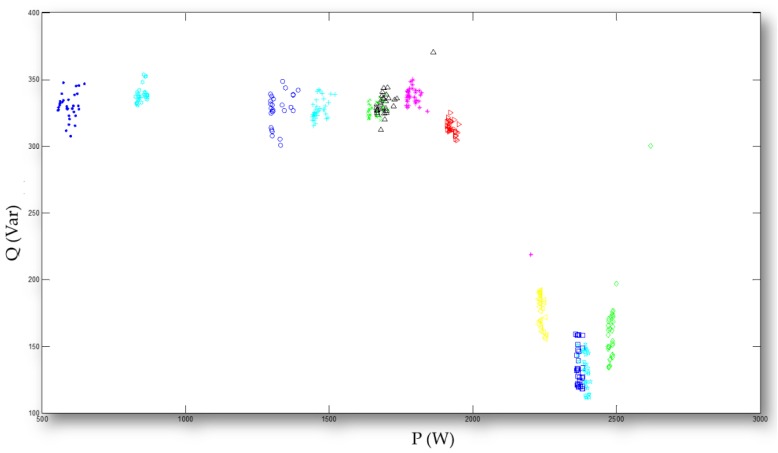
Feature space being of P and Q. The entire space is addressed by the configured feed-forward ANN, with/without the parallelism, in this work. A total of 12 load combinations including the scenarios in which the four electrical appliances are the mutually exclusive load combinations that need to be addressed.

In this experiment, a total of 960 data instances are examined. Figure 10 shows the feature space addressed by the configured feed-forward ANN evolved in parallel. The feature space is of extracted electrical features P and Q. Parameters specified for the parallel GA used in this experiment to evolve/train the configured feed-forward ANN run over the 960 data instances for load classification in the NILM are below. The population size, *pop_size*, is 1,000. In addition, a random initial population is created with a uniform distribution, where the uniform distribution is in the initial population range [−10, 10] and where the total length of each chromosome is 179 (= (2 + 1) × 25 + (25 + 1) × 4). The stochastic uniform selection is used, and, for this proportional selection procedure, the algorithm scales raw fitness values based on the rank of individuals instead of their raw fitness value. Moreover, the elitist strategy is conducted. A total of top (0.05 × *pop_size*) individuals/chromosomes in the existing population are guaranteed to survive to the next population. The scattered crossover operator is used. The crossover fraction of the population is 0.65. The gaussian mutation operator is used. The mutation rate is 0.01. The maximum number of generations is 500. Lastly, the fitness function is clarified as –*E*, where *E* is the adopted cost function of the configured feed-forward ANN.

**Figure 11:**
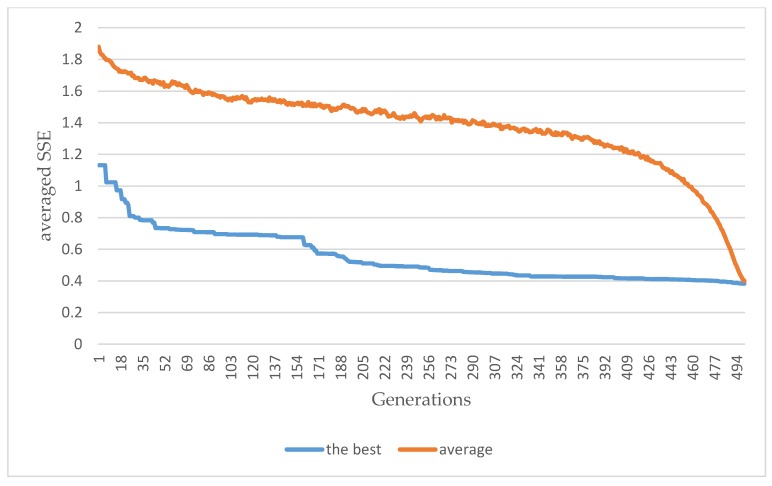
Evolution trajectory obtained by the parallel GA for the configured feed-forward ANN in this experiment. The algorithm determines the value of fitness as the inverse of *E*: the lower the error is, the higher the fitness is.

In this experiment, the evolutionary trajectory obtained by the parallel GA is shown in Figure 11. As shown in Figure 11, the average SSE obtained was 0.38. The trained weight coefficients by the parallel GA for the configured feed-forward ANN are shown in Appendix A. The averaged SSE of 0.38 was obtained, as shown in Figure 11. Table 1 shows the load classification results obtained by the configured feed-forward ANN trained/evolved by the parallel GA. In Table 1, the load classification rate is defined and computed, for electrical appliances, as Equation (2).

(2) $$\sum_{t\in T}x_{t}^{i}$$

In Equation (2), *T* in *T* denotes the total number of time slices in a day and *x^i^_t_* (*t* ∈ *T* = {1, 2, 3, ..., *t*, ...*T*}) is 1 if electrical appliance *i* is correctly identified by the NILM. Otherwise, it is 0 (*x^i^_t_* can be used to statistically estimate kWh of electrical appliance *i*).

**Figure 12:**
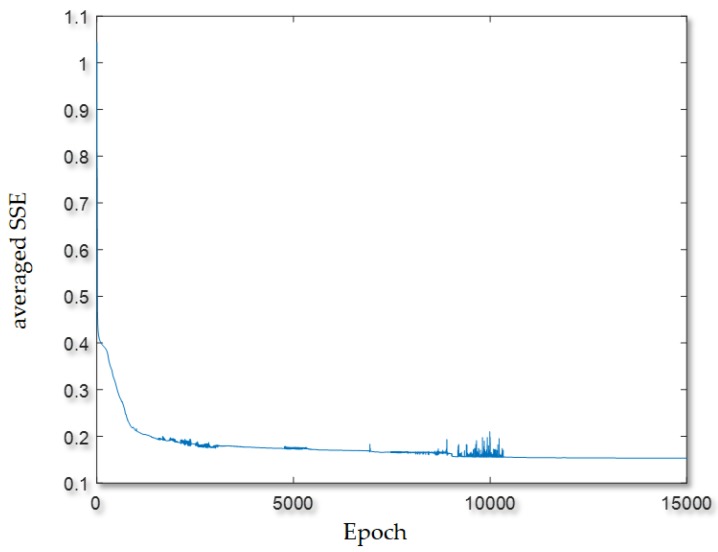
Training trajectory obtained by the widely-used GD algorithm for the feed-forward ANN in this experiment. This plot implies that only 15,000 feed-forward ANNs differing in their trainable weight coefficients were examined for NILM in this work.

In Table 1, the computation time consumed by the parallel GA-embodied ANN is also shown, and it is 6.94 min. A total of 501,000 function evaluations/routines meaning that totally 501,000 feed-forward ANNs differing in their weight coefficients are examined and done in a parallel execution where the number of workers started up and used on the Acer Predator G3-710 Intel^®^ Core^TM^ i7-6700 CPU (@ 3.40 GHz) desktop computer (RAM: 16 GB) for parallel computing is 4. As shown in Table 1, in this experiment, the configured feed-forward ANN is also trained by the widely-used GD algorithm, where an averaged SSE of 0.17 was achieved (the achieved load classification rates are unacceptable), as shown in Figure 12. It is also meta-heuristically trained/evolved by a standard GA, where its training is very time-consuming. As shown in Table 1, the HEMS utilizing the configured feed-forward ANN meta-heuristically trained/evolved by the parallel GA (a parallel GA is used to meta-heuristically train adjustable weight coefficients of an architecture-given feed-forward ANN in parallel) gets an acceptable level of NILM accuracy. The configured feed-forward ANN trained by the widely-used GD algorithm is easily fooled by local minima and the configured feed-forward ANN meta-heuristically trained/evolved by a standard GA instead of the widely-used GD algorithm is highly consumed in computation time (the undertaken NILM in this experiment throws an acceleration of up to 48× achieved with four workers on the computer for parallel computing).

**Figure 13:**
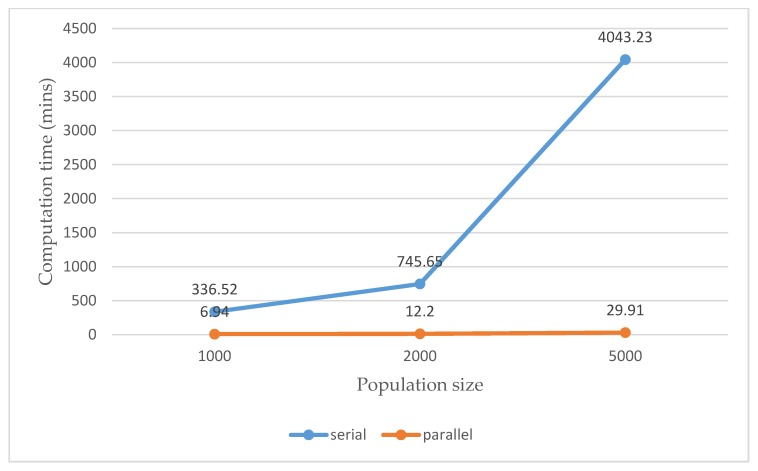
Computation time by the standard GA vs. the parallel GA in this experiment. The parallel GA against the standard GA has, in computation time, an acceleration of up to 135× (=4043.23/29.91) achieved.

In order to improve the load classification results, by the ANN evolved by the parallel GA against the GA, in Table 1, the parallel GA starts with 5000 randomly-generated initial chromosomes to meta-heuristically train the feed-forward ANN, since it is usually argued that a small population size could guide a GA to poorly search for the global optimal solution. The resulting averaged SSE is 0.25 (the load classification rates are improved) and the elapsed time is 29.91 min. More workers can be configured and used to substantially accelerate the algorithm, as shown in Table 2. The parallel GA starting with 5000 randomly-generated initial chromosomes (the population size: 5000) takes 29.91 min. The computation time by the standard GA versus the parallel GA in this experiment increases exponentially, as shown in Figure 13. The parallel GA considering a population size of 5000 specified meta-heuristically evolves the configured feed-forward ANN for load classification with an acceleration of up to 135 × achieved against the standard GA, in order to improve the optimization accuracy and substantially reduce the execution time. As seen in Figure 13, it took the standard GA a very long time to evolve the configured feed-forward ANN.

**Figure 14:**
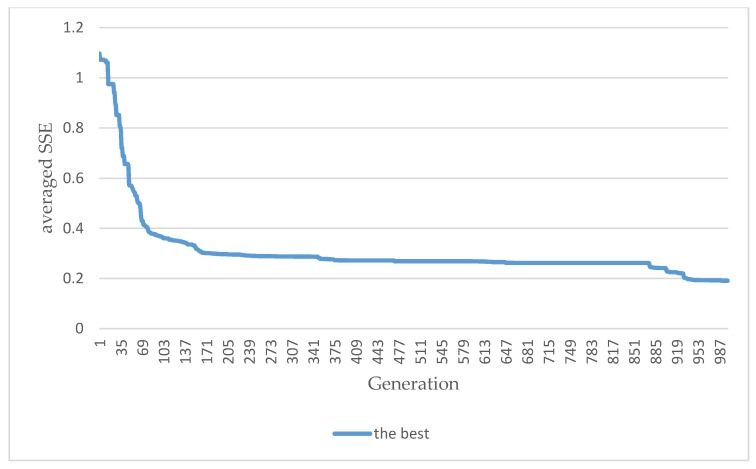
Trajectory of the parallel GA used to meta-heuristically train the feed-forward ANN. In total, 7,507,500 feed-forward ANNs differing in their trainable weight coefficients are considered and they are examined through parallel computing, and the algorithm takes 85.21 mins for the evolution.

In the last part of this experiment, the feed-forward ANN is meta-heuristically trained by a parallel GA where the population size specified is 7500 and the maximum number of generations specified is 1000. Figure 14 shows the evolutionary trajectory of the parallel GA. The resulting averaged SSE shown is 0.1909. The optimal weight coefficients trained by the parallel GA for the configured feed-forward ANN used for load classification of NILM in this work are shown in Appendix B. In order to quantitatively evaluate the parallel GA for its performance in terms of convergence accuracy, this experiment executes the algorithm for 10 trials. Wherein, the resulting mean and standard deviation are 0.2341 and 0.0452, respectively. Consequently, the load classification rates obtained from the best trial of the 10 runs of the algorithm in this experiment are outlined in Table 3. As shown in Table 3 and compared with the overall load classification rate that was obtained in Reference [12], the overall load classification rate in this work is improved by 3.46%. On-line load monitoring is performed on-site, as the feed-forward ANN evolved by the parallel GA has been well trained through the off-line training process.

## 4. Discussion

This work develops and applies a parallel GA to meta-heuristically evolve a feed-forward ANN in order to achieve its optimal adjustable parameters, optimal trainable weight coefficients including biases, and speed up the evolutionary process for evolving it for load classification in NILM conducted as a part of an HEMS for DSM. A fully-nonintrusive load classification in NILM in this work is suggested and considered. The detailed NILM pipeline in this work is depicted in Figure 3, and it is referenced in the experiment conducted in a real residential environment. As demonstrated in Section 3, shown in Figure 13, and reported in Table 3, the feasibility and effectiveness of the undertaken NILM that throws an improved overall load classification rate of 95.13% under an achieved acceleration of up to 135× for its training have been experimentally validated. The immersion of a parallel GA into a feed-forward ANN used as a load classifier of NILM to non-intrusively identify electrical appliances for traces of their operation is needed, as (i) the feed-forward ANN used and trained by the conventional GD algorithm is easily fooled by local minima and (ii) the sequential optimization process for meta-heuristically training the feed-forward ANN evolved through evolutionary computing is extremely computationally intensive. To load classification in the NILM, more electrical features would be extracted through feature engineering of creating features from raw data, and investigated. Usually, the better the characterization of data extracted from electrical appliances, the higher the performance (predictive power) of AI for load classification. The developed parallel GA-embodied ANN in this work is a revision of the methodology that was done in Reference [12]. In addition, it is a preliminary design of NILM toward deep learning. Deep NNs in deep learning have become more attractive AI than ANNs in recent years. In the future, deep NNs having many multiple hidden layers will be investigated and used, over fog-cloud analytics [2,37,38], by the developed parallel GA-embodied ANN for NILM in DSM, where distributed Graphics Processing Unit (GPU) computing can be configured to run parallel evolutionary computing to meta-heuristically train a deep NN across multiple CPUs and GPUs on a single computer or a cluster of multiple computers.

## 5. Conclusions

NILM, which is a more cost-effective load monitoring approach than traditional intrusive load monitoring approaches and a part of HEMS for DSM, is capable of disaggregating aggregated electric energy consumption into appliance-level power consumption based on electrical features extracted from electrical appliances and modeled by AI methodology. A feed-forward ANN trained by the widely-used GD algorithm can be used to address NILM as a load classification task, but it is easily fooled by local minima. A GA, which is a global search algorithm against the widely-used GD algorithm, can be used to meta-heuristically train/evolve a feed-forward ANN run over massive training samples for fitness evaluation, but it is very time-consuming. In this situation, it is necessary to parallelize the algorithm and, thus, substantially reduce the computation time by speeding up the algorithm. Hence, in this work, a parallel GA-embodied ANN for NILM in an HEMS has been developed in consideration of parallel computing where a GA used to meta-heuristically train/evolve a feed-forward ANN run over massive training samples for fitness evaluation for load classification (off-line training) of NILM is parallelized and accelerated. On-site collected data learned by the developed parallel GA-embodied ANN for NILM are labeled through sensor fusion. Fully-nonintrusive load classification in NILM in this work is suggested and considered. The experimental results reported in this work show the feasibility and effectiveness of the developed parallel GA-embodied ANN for NILM in the HEMS in this work. In addition, the undertaken NILM throwing an achieved acceleration of up to 135× is shown. Lastly, the improvement in the overall load classification rate that is obtained in this work and compared with that of the work that was done in the previous study is shown.

The parallel GA-embodied ANN in this work is a preliminary design of NILM towards deep learning. By definition, deep learning is the applications of multi-neuron, multi-layer ANNs always treated as a black box [49] to perform learning tasks, including regression, classification, clustering, auto-encoding, and others, through a series of many layers containing initiated neurons for each of them and combining all features learned by their previous layers across data. ANNs having this kind of many-layer structures such as two or more hidden layers, building up a hierarchy of ever more complex and abstract concepts from data are called deep NNs [46,47,49]. Deep NNs in deep learning have become more attractive AI than ANNs in recent years. In the future, deep NNs having many multiple hidden layers will be investigated and used over fog-cloud analytics [2,37,38] by the developed parallel GA-embodied ANN for NILM in Demand-Side Management (DSM), where distributed GPU (Graphics Processing Unit) computing can be configured to run parallel evolutionary computing to meta-heuristically train a deep NN across multiple CPUs and GPUs on a single computer or a cluster of multiple computers.

## Figures and Tables

**Table 1:** Load classification rates obtained in this experiment.

Electrical Appliances	Load Classification Rates by Different AI Approaches (%)			Improvement in Load Classification Rate (%)
	the configured ANN trained by the GD algorithm	the configured ANN evolved by a standard GA	the configured ANN evolved by the parallel GA	
electric rice cooker	94.06	74.06	85.62	−8.44
electric water boiler	44.27	79.17	92.81	+48.54
steamer	55.73	88.54	90.10	+34.37
TV	47.19	89.69	83.23	+36.04
**Computation time (mins)**	6.68	336.52 ^2^	6.94	-
**Improvement in computation time ^1^ (mins)**	-	-	−329.58	-

^1^ The undertaken NILM in this experiment throws an acceleration of up to 48× achieved with four workers started up and used on the Acer Predator G3-710 Intel^®^ Core^TM^ i7-6700 CPU (@ 3.40 GHz) computer (RAM: 16 GB) for parallel computing. ^2^ A total of 501,000 function evaluations were done in a serial execution. In total, 501,000 feed-forward ANNs differing in their weight coefficients were examined.

**Table 2:** Improved load classification rates by the parallel GA-embodied ANN, in this experiment, considering 2,505,000 feed-forward ANNs differing in their trainable weight coefficients.

	**Population Sizes** (the maximum number of generations: 500)				
	1000 (501,000 function evaluations)	2000 (1,002,000 function evaluations)		5000 (2,505,000 function evaluations)	
	**the configured ANN evolved by the standard GA**				
**Computation time** ^1^ **(mins)**	336.52	745.65		4043.23	
**Averaged** Sum of Squared Errors **(SSE)**	0.40	0.40		0.47	
	**the configured ANN evolved by the parallel GA**				
**Computation time (mins)**	6.94	12.20		29.91	
**Averaged SSE**	0.38	0.36		0.25	
	**Load classification rates by the configured ANN evolved by the different types of GA considering the different sizes of population (%)**				**Improvement in load classification rate (%)**
**Electrical appliance**	**the configured ANN evolved by the standard GA** (population size: 1000)		**the configured ANN evolved by the parallel GA** (population size: 5000)		
electric rice cooker	74.06		83.75		+9.69
electric water boiler	79.17		94.69		+15.52
steamer	88.54		90.83		+2.29
TV	89.69		98.96		+9.27

^1^ The computation time increases exponentially. Meta-heuristically training/evolving the configured feed-forward ANN in a serial/sequential execution is extremely computationally intensive.

**Table 3:** Load classification rates obtained by the parallel GA embodied and used to meta-heuristically train the feed-forward ANN in this experiment.

**Electrical Appliances**	**Load Classification Rates by the GA-Embodied Feed-forward ANN (%)** (the maximum number of generations: 1000) (the population size: 7500)
electric rice cooker	90.00
electric water boiler	95.52
steamer	95.73
TV	99.27
Overall load classification rate (%)	95.13
**Overall load classification rate improved ^1^ (%)**	+3.46

^1^ The overall load classification rate that is obtained in this work is compared with that of the work that was done in Reference [12].

## Appendix A

*v_qj_*

[*w_iq_*]*^T^*

## Appendix B

*v_qj_*

[*w_iq_*]*^T^*
